# Metabolic markers during pregnancy and their association with maternal and newborn weight status

**DOI:** 10.1371/journal.pone.0180874

**Published:** 2017-07-27

**Authors:** Otilia Perichart-Perera, Cinthya Muñoz-Manrique, Angélica Reyes-López, Maricruz Tolentino-Dolores, Salvador Espino y Sosa, Ma. Cristina Ramírez-González

**Affiliations:** 1 Nutrition and Bioprogramming Department, Instituto Nacional de Perinatología Isidro Espinosa de los Reyes, Mexico City, Mexico; 2 Clinical Research Subdirection, Instituto Nacional de Perinatología, Mexico City, Mexico; East Tennessee State University, UNITED STATES

## Abstract

**Background/Aims:**

Obesity during pregnancy increases the risk of adverse clinical outcomes and is associated with low-grade chronic inflammation. We describe maternal metabolic risk and inflammation by maternal weight status, and evaluate the association of metabolic and inflammatory markers with birthweight in a group of pregnant Mexican women.

**Methods:**

This study derived from a prospective cohort of healthy pregnant women <14 weeks of gestation, receiving prenatal care at National Institute of Perinatology (Mexico, 2009–2013). Metabolic and inflammatory markers were measured in maternal serum in all three pregnancy trimesters (1st: 11.42±1.7; 2nd: 21.06±2.4; 3rd: 32.74±2.3 weeks). Pregestational weight was self-reported, and body mass index (BMI) was calculated. Gestational weight gain was evaluated in the third trimester. Newborn´s weight was measured at birth. We carried out correlations, general mixed linear model and regression analyses, based on pregestational weight (self-reported), body mass index (BMI), gestational weight gain (evaluated in the third trimester) and newborn weight (measured at birth).

**Results:**

Of the 177 women included in the study (mean age = 26.93±8.49), thirty-eight percent (n = 67) were overweight or had obesity, and 32.8% (n = 58) showed excessive gestational weight gain. We found insulin, lipids (including total cholesterol, LDL-cholesterol, HDL-cholesterol, and triglycerides-TG), leptin and interleukin 1b (IL-1b) all increased significantly (p<0.05) during pregnancy. Pregestational maternal weight status altered longitudinal concentrations of insulin, leptin, adiponectin, TG and C reactive protein. Excessive gestational weight gain was associated with higher maternal insulin in the third trimester (p<0.05). Early pregnancy leptin and TNFα were determinants of birthweight in women with normal weight, but not in overweight or obese women.

**Conclusions:**

Maternal weight status affected the concentrations of insulin, leptin, adiponectin, triglycerides and C reactive protein throughout pregnancy. The role of early leptin and TNFα in fetal growth need further study given the association was only observed in normal weight women. This study presents data distribution of metabolic and inflammatory markers of normal weight and overweight/obese women that did not develop GDM, preeclampsia nor macrosomia.

## Introduction

Obesity is a public health problem in Mexico as it is around the world [1]. As of 2016, 75.6% of Mexican women of reproductive age are considered to be overweight or obese [2]. For prospective mothers, entering pregnancy with obesity increases the risk of gestational diabetes mellitus (GDM), preeclampsia, miscarriage, C-section and hemorrhage, among other complications. Newborns from obese mothers have a higher risk of being classified as large for gestational age (LGA), macrosomic (>4000 g at birth), or small for gestational age (SGA), as well as having a higher risk of developing obesity in later life [3,4].

Metabolic abnormalities may result from excessive adipose tissue accumulation because of low-grade chronic inflammation linked to macrophage infiltration and the release of many adipokines. These adipokines are bioactive compounds that participate as mediators in many metabolic pathways, and affect energy substrate use in both fetus and offspring [5,6]. Metabolic programming of obesity and chronic diseases due to hormonal and nutritional changes during pregnancy have been well documented in animal and human studies [4,7]. Maternal hyperinsulinemia, hyperleptinemia, hipoadiponectinemia and inflammation are associated with excessive nutrient transport at the placental level, which results in increased fetal growth and programs the fetus for future disease [8].

Maternal glucose and triglyceride levels have been positively associated with birth weight, even in women without GDM [9,10]. Likewise, maternal and cord-blood leptin and adiponectin levels have been associated with higher birthweight and higher adiposity in newborns [11,12]. However, mixed results (including inverse trends) exist regarding the associations between leptin and birthweight [13]. Inflammation has also been associated with birthweight in some studies [14,15]. In women without GDM, the main determinants of having an LGA infant appear to be maternal obesity, maternal weight gain and leptin levels [12,16,17].

Very few prospective studies have described insulin, lipid, adipokine and inflammatory changes during pregnancy in relation to pregestational obesity or excessive gestational weight gain [12,13,18]. The aim of this study was to describe maternal metabolic risk and inflammation given weight status before and during pregnancy, as well as to evaluate the association of metabolic and inflammatory markers with birthweight in a group of pregnant Mexican women.

## Materials and methods

### Study design and subjects

This descriptive study derives from a prospective cohort of pregnant women that were followed at the National Institute of Perinatology (Mexico City) from 2009 to 2013. The study was approved by the Ethics Committee and the Research Committee at the Instituto Nacional de Perinatología in Mexico City, under project number 08311. We obtained signed informed consent from all selected participants, and in the case of adolescents (<19 years old), both parents and individuals gave consent.

All pregnant women who came for prenatal care at the Institute were evaluated for possible participation. Women at <14 weeks of gestation were selected consecutively, unless they had multiple pregnancies, previous DM, autoimmune, renal or hepatic disease, or if they were taking medications that affect metabolism (steroids, insulin, and metformin, among others). Women with controlled subclinical hypothyroidism (thyroid profile within ranges) were included. For this descriptive analysis, women were excluded due to: GDM (one altered value in a 2 hour 75 g oral glucose tolerance test at any point during pregnancy) [19]; preeclampsia (blood pressure ≥140/90 mmHg and proteinuria >300 mg/24 hour after 20 weeks of gestation) [20]; ≤ 2 blood measurements in two different trimesters; fetal malformation; and/or prednisone use during pregnancy.

Women were followed until the end of pregnancy, and nutritional assessment and blood sample collection were carried out in each trimester.

### Maternal obesity and gestational weight gain

We measured weight to the nearest ±0.1 kg, with women wearing light clothing and no shoes, using a calibrated digital scale (TANITA BMB-800), and height to the nearest 0.1 cm using a digital stadiometer (SECA 264). Pregestational weight was self-reported and we calculated pregestational BMI. Women were classified as overweight or obese if their pregestational BMI was ≥25 kg/m2[21].

Gestational weight gain was corrected by length of gestation on the last visit (third trimester). First trimester weeks of gestation (13 weeks) were subtracted from the last visit weeks of gestation and multiplied by the recommended weight gain range for each BMI category, following the Institute of Medicine guidelines [22]. Finally, first trimester weight gain range (0.5–2.0 kg) was added to the previous range. Women were classified as having adequate, insufficient or excessive weight gain for their gestational age.

### Parity

Women were classified as nulliparous if they have never given birth.

### Gestational age

Gestational age was estimated by ultrasound during the first trimester. In cases where no ultrasound was available, we calculated weeks of gestation according to the last menstrual period.

### Metabolic blood measurements

We collected a fasting peripheral venous blood sample each trimester and centrifuged them at 3000 rpm to obtain serum, which was then kept at -70°c until laboratory analysis. Serum glucose concentrations (Trinder method using glucose oxidase reactive) [23], total cholesterol (enzymatic colorimetric-Diasys) (TC), HDL- cholesterol (HDL-C) (enzymatic colorimetric-Diasys), and triglycerides (TG) (enzymatic colorimetric-Diasys) were analyzed using an automatic analyzer (LORY 2000, Diasys; coefficient of variation <5%). We used the Friedewald formula to calculate LDL- cholesterol (LDL-C) [24].

C-reactive protein (CRP), insulin, and interleukins were measured by quimioluminiscence (Immulite 1000; Siemens Health Care Diagnostic, IL, U.S.A) with the following detection ranges: CRP 0.1 mg/L, insulin 2 μIU/mL, IL1b 2–1000 pg/mL, IL6 2–1000 pg/mL and TNF-α 1.7–1000 pg/mL. Interassay coefficients were <10%. Leptin and adiponectin concentrations were quantified with the Enzyme-Linked ImmunoSorbent Assay (ELISA), sandwich type (R&D Systems, Minneapolis, U.S.A.). Calibration curves (log/log curve fit) were developed for adiponectin from 0–250 ng/mL and for leptin from 0–1000 pg/mL. Interassay variation coefficients were <10%.

Homeostatic model assessment was computed (HOMA-IR) using fasting glucose and fasting insulin concentrations [25].

### Birth weight

Newborn birthweight was measured at birth by the medical staff and we collected data from the medical record.

### Statistical analysis

We obtained descriptive statistics (Mean±SD; Median: 25–75°) and frequencies for all variables, then carried out partial correlations (Spearman) for metabolic blood measurements and weight variables. We did an exploratory analysis of the association between metabolic markers (glucose, insulin and lipids) with adipokines and CRP, as the latter are important mediators of fetal growth and exert an effect on substrate utilization. Non normal variables were naturally log transformed. We ran general mixed linear models to evaluate the effect of pregnancy and being overweight/obese before pregnancy on changes in metabolic and inflammatory markers. One model was run for each marker. For subgroup analysis, we stratified the models by parity, age group and gestational weight gain category. Categorical data were analyzed with Chi square test/Fisher test. To evaluate determinants of birthweight, we ran crude and adjusted linear regression models, adjusting by gestational age at birth. Statistical analyses were carried out using Statistical Package for the Social Sciences (SPSS) software, version 20.0 (Chicago, IL).

## Results

Of a total 298 women who met the inclusion criteria and were accepted to participate, 79.1% (n = 236) continued the study until the third trimester. Because this was a descriptive analysis in healthy women, 59 individuals were eliminated due to: GDM and/or preeclampsia (n = 46), ≤ 2 blood measurements (n = 9); fetal malformation (n = 1); and/or prednisone use during pregnancy (n = 3). In the end, data from 177 women was included.

Demographic characteristics are shown in Table 1. Mean age was 26.93±8.49 years of age (range: 13–44 years). Adolescents (≤19 years old) represented 27.1% (n = 48) of the total sample of women. At baseline, medication use was low (7.3%, n = 13), with the most common reported medications being antibiotics (n = 3), thyroid medication (n = 3), and low dose aspirin (n = 2). At the end of pregnancy, medication use increased (31.6% of women, n = 56), and the most frequent reported medications were aspirin (n = 19), thyroid medication (n = 4) and antacids (n = 6). Mean gestational ages in the first, second and third trimester of pregnancy were 11.42±1.7 weeks, 21.06±2.4 weeks and 32.74±2.3 weeks, respectively.

**Table 1:** Baseline characteristics of women by pregestational weight status.

	All women (n = 177)	Normal weight/underweight (BMI<25) (n = 109)	Overweight/obesity (BMI weight) (n = 67)
**Gestational age (first trimester) (weeks) ^a^**	11.42±1.72	11.22±1.76	11.73±1.66
**Age (years)^a^**	26.9 ± 8.4	25.2 ± 8.6	29.5 ± 7.5**
**Adolescents (<19 years) (%) (n)**	27.1% (n = 48)	36.7% (n = 40)	11.9% (n = 8)*
**Weight (kg) ^a^**	59.72± 13.29	51.92 ±6.95	72.30±11.28**
**Height (kg) ^a^**	156.53±5.62	156.25±5.86	156.92±5.22
**Pregestational BMI (kg/m^2^) ^a^^b^**	24.4 ± 5.0	21.2 ± 2.1	29.4 ± 4.2
**Medication use (%) (n)**	7.3% (n = 13)	3.6% (n = 4)	13.4% (n = 9)*
**Nulliparous (%) (n)**	58.8% (n = 104)	70.6% (n = 77)	40.3% (n = 27)*
**Education level (high school or higher) (%) (n) ^a^**	75.6% (n = 133)	74.3% (n = 81)	77.3% (n = 51)
**Married or lived with a partner (%) (n)**	63.8% (n = 113)	55% (n = 60).	77.6% (n = 52)*
**Housewives (%) (n)**	69.4% (n = 123)	64.2% (n = 70)	79.1% (n = 53)

^a^ Mean±SD

^b^ Pregestational weight was not available (n = 1).

*Chi square/Fisher exact test

**Student´s t test. p<0.05

Pregestational overweight and obesity prevalence was 38.1% (n = 67). Pregestational BMI was higher in adults, multiparous women, and in women who lived with a partner (p<0.001). Mean gestational weight gain during the third trimester was 9.73± 4.84 kg. Adequate gestational weight gain was observed in 39.5% (n = 70) of women; while 32.8% (n = 58) of women were classified with excessive weight gain. Overweight/obese women had lower weight gain when compared to normal weight women (8.27±4.60 kg vs 10.64±4.79 kg, p = 0.001). Weight gain classification was similar between normal weight and overweight/obese women (p = 0.448).

We carried out correlations between glucose, insulin and lipids with adipokines and CRP, and between metabolic markers with pregestational BMI and newborn weight (Table 2). TC and HDL-C correlated inversely with cytokines at different times. Insulin and HOMA-IR correlated positively with leptin, adiponectin and CRP. Pregestational BMI correlated with many metabolic and inflammatory markers, while gestational weight gain did not correlate with any marker. First trimester leptin and TNFα, and second trimester glucose were the only markers that correlated with birthweight.

**Table 2:** Significant correlation coefficients of the association between metabolic and inflammatory markers and between maternal and newborn weight with metabolic and inflammatory markers.

		FIRST TRIMESTER	SECOND TRIMESTER	THIRD TRIMESTER
**INSULIN**	Leptin	—	0.254**	0.184*
	Adiponectin	—	—	-0.214**
	CRP	0.255**	0.306***	0.266***
	TNFα	0.235**	—	—
**HOMA-IR**	Leptin	—	0.220**	0.212**
	Adiponectin	—	—	-0.174*
	CRP	—	0.228**	0.236**
**TOTAL CHOLESTEROL**	Leptin	—	0.209**	—
	TNFα	-0.288**	—	-0.168*
	IL-1b	-0.243**	—	-0.165*
	IL-6	-0.258**	—	-0.235**
**HDL-CHOLESTEROL**	Leptin	—	0.188**	—
	IL-1b	-0.217**	—	-0.216**
	IL-6	—	-0.191**	-0.331***
	TNFα	-0.357***	—	—
**TRIGLYCERIDES**	Adiponectin	—	-0.162*	—
**GLUCOSE**	Leptin	—	0.183**	—
	TNFα	-0.310***	—	—
	IL-1b	-0.235**	—	-0.205**
**PREGESTATIONAL BMI**	Insulin	0.432***	0.406***	0.370***
	Leptin	0.270**	0.237***	0.192**
	TG	0.256**	0.178**	0.164*
	CRP	0.343***	0.294***	0.280***
	Glucose	—	0.160*	—
**BIRTHWEIGHT**	Leptin	0.235**	—	—
	TNFα	-0.196*	—	—
	Glucose	—	0.174*	—

Spearman correlation. Only statistical significant correlations are shown.

Statistical significance

*p≤0.05

**p≤0.01

***p≤0.001

**Fig 1:**
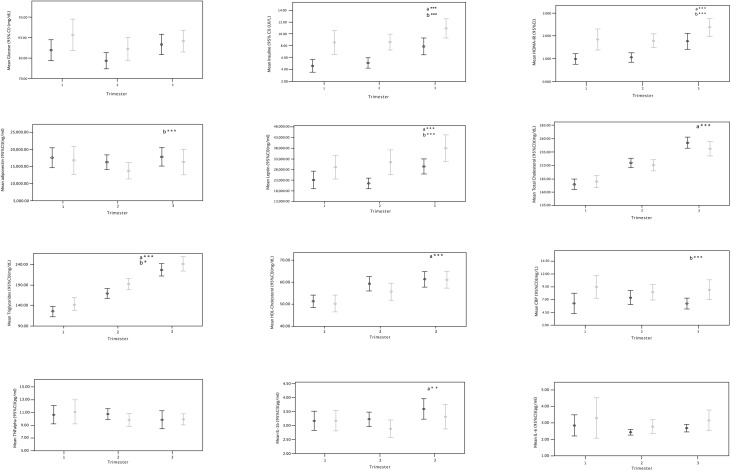
Metabolic and inflammatory markers during pregnancy and by pregestational weight status.

Significant increases in insulin, HOMA-IR, leptin, TC, LDL-C, HDL-C, TG and IL1b were observed during pregnancy (p<0.05). Adiponectin levels were maintained (Fig 1). When maternal weight status was added to the general linear model, being classified as overweight/obese modified the longitudinal concentrations of insulin, HOMA, leptin, adiponectin, TG and CRP (p<0.05) (Fig 1, Table 3).

**Table 3:** General mixed linear models evaluating the effect of pregnancy and pregestational overweight/obesity on metabolic and inflammatory markers.

		B	95% CI	p value
**Insulin**	Time	0.237	0.179, 0.294	**<0.001**
	Overweight/Obesity	0.531	0.360, 0.703	**<0.001**
**HOMA-IR**	Time	0.021	0.015, 0.027	**<0.001**
	Overweight/Obesity	0.047	0.027, 0.066	**<0.001**
**Adiponectin**	Time	-0.025	-0.091, 0.041	0.455
	Overweight/Obesity	-0.082	-0.353, -0.010	**0.038**
**Leptin**	Time	0.154	0.079, 0.228	**<0.001**
	Overweight/Obesity	0.272	0.109, 0.437	**0.001**
**TNFα**	Time	-0.036	-0.078, 0.006	0.094
	Overweight/Obesity	-0.014	-0.108, 0.078	0.753
**IL1b**	Time	0.058	0.014, 0.103	**0.010**
	Overweight/Obesity	-0.070	-0.179, 0.038	0.206
**IL-6**	Time	0.125	-0.011, 0.063	0.173
	Overweight/Obesity	0.094	-0.006, 0.195	0.066
**CRP**	Time	0.055	-0.026, 0.138	0.186
	Overweight/Obesity	0.395	0.173, 0.616	**<0.001**
**Glucose**	Time	0.003	-0.010, 0.018	0.613
	Overweight/Obesity	0.028	-0.001, 0.059	0.06
**Total Cholesterol**	Time	36.07	32.68, 39.46	**<0.001**
	Overweight/Obesity	-4.81	-17.66, 8.03	0.462
**Triglycerides**	Time	52.87	47.74, 58.00	**<0.001**
	Overweight/Obesity	18.43	1.91, 34.95	**0.029**
**HDL-Cholesterol**	Time	4.86	3.50, 6.22	**<0.001**
	Overweight/Obesity	-1.64	-5.67, 2.38	0.424

General mixed linear models were performed for each marker. Statistical significance was considered when p value<0.05 Time: Measurements done at first, second and third trimesters of pregnancy. Overweight/Obesity: Presence of overweight/obesity classified with pregestational BMI (BMI≥25.0 kg/m^2^).

When the models were stratified by parity, age group (adolescents vs. adults) and gestational weight gain, the effects and direction of the associations were maintained, but in some cases statistical significance was lost.

**Fig 2:**
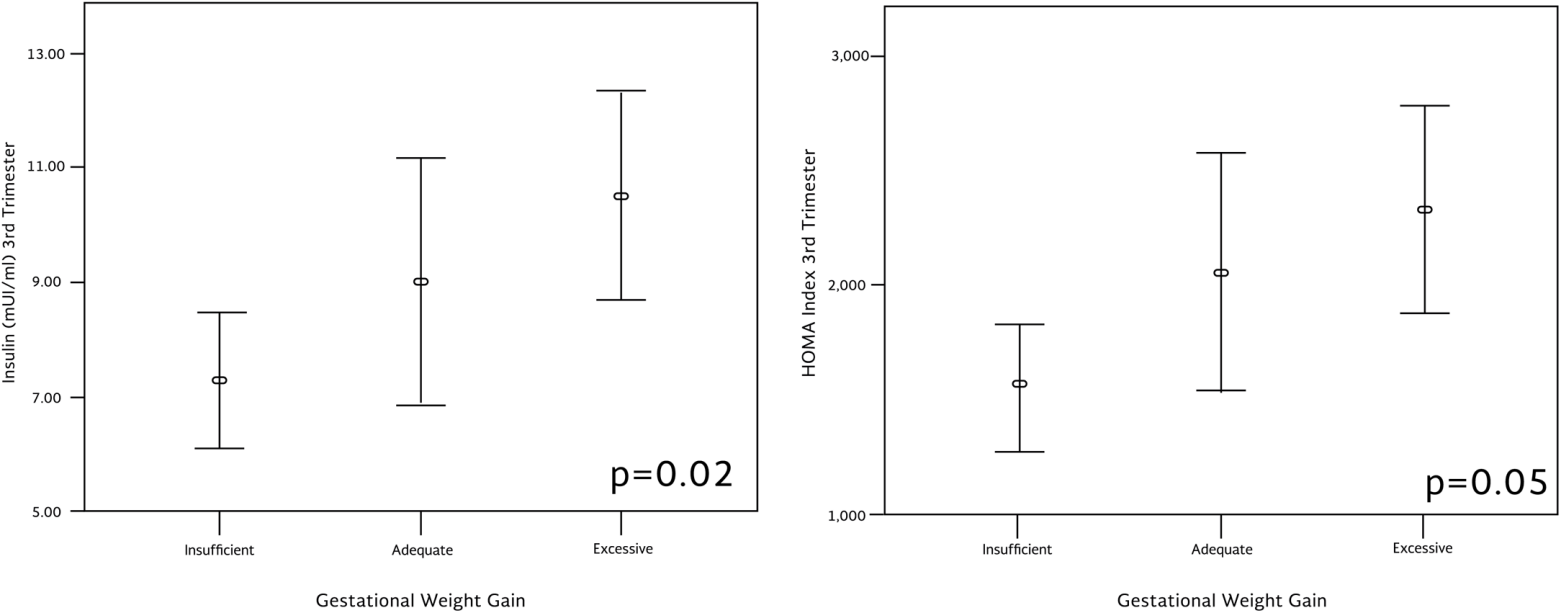
Insulin concentration and HOMA during the third trimester according to gestational weight gain.

Women who had excessive gestational weight gain showed significantly higher insulin levels (p = 0.02) and higher HOMA-IR although the latter was non-significant (p = 0.05) compared to women with adequate or insufficient gestational weight gain (Fig 2).

Mean gestational age at birth was 38.20±2.05 weeks of gestation; 18.07% (n = 32) of the births were preterm. Mean birthweight was 2959.33±460.41 g. Higher mean birthweight was observed in women who had excessive gestational weight gain (3156.75±403.96 g) compared to those who had adequate gestational weight gain (2861.05±478.79 g) and insufficient weight gain (2904.93±422.36 g) (p = 0.001).

No differences in gestational age or birthweight were observed in relation to pregestational obesity.

Individual linear regression models, stratified by BMI category, showed first trimester leptin and first trimester TNFα were significantly associated with birthweight, but only in normal weight women (Normal weight: leptin B = .007 95%CI: .002, .011, p = 0.005 and TNFα B = -14.99 95%CI: -29.43,-0.543, p = 0.042; Overweight/Obesity: leptin B = .002, 95%CI: -.005, .009, p = 0.531 and TNFα B = 1.09, 95%CI: -20.35, 22.54, p = 0.918). Models were adjusted by gestational age at birth. Glucose (2nd trimester) was not a significant determinant of birthweight.

The changes in leptin, TNFα, and glucose (3rd trimester concentrations minus 1st trimester concentrations) were not significant determinants of birthweight.

In women with low birthweight newborns, first trimester maternal leptin concentrations and second trimester IL-1b were also lower than in women with normal weight newborns (p = 0.011, p = 0.032, respectively). We did not find any differences in maternal concentrations of metabolic or inflammatory markers in preterm infants.

## Discussion

Very few studies with prospective design have evaluated serum adipokines and inflammation during pregnancy in healthy women. This study confirmed that insulin, leptin and lipids increased significantly throughout pregnancy. Women who were overweight/obese at the beginning of pregnancy showed higher insulin, HOMA, leptin, TG, and CRP levels throughout pregnancy, and showed a decrease in adiponectin. Currently, there are no accepted limits for lipid increase during pregnancy, due to the high variability observed between women. Excessive circulating lipids may result in excessive newborn or child adiposity [26,27], but risk is difficult to assess. Lipid data in this study are comparable to the reported ranges in normal pregnancy in other populations [28,29], and represent the first longitudinal data reported in Mexican women. TG were higher throughout pregnancy in overweight/obese women, but were not related with birthweight. Some studies have found that third trimester TG levels are determinants of birthweight [30].

An important finding was the association of early pregnancy maternal leptin and TNFα with birthweight. Moreover, this association was only present in women that started pregnancy with normal weight. The association of early pregnancy leptin with birthweight has been reported before [12,31], although longitudinal studies are scarce. In the study by Walsh.et.al. (2014), early maternal leptin was a significant predictor of infant size at birth, as well as third trimester maternal leptin. Models were adjusted by pregestational BMI, but included all women [11]. In the study by Misra et.al. (2013), an inverse association between net leptin change during pregnancy and birthweight, in overweight/obese women was reported, which is different to what we observed [32]. The role of leptin on fetal growth is influenced by many physiologic factors that appear to be different in normal weight and overweight/obese women. The lack of consistency in these associations may be explained by biological mechanisms that need to be studied. It seems that maternal-fetal communication and placental signaling mechanisms are different in overweight/obese women compared to those of normal weight.

Normal pregnancy has been associated with mild elevations of anti and pro-inflammatory cytokine levels [18,33]. In our study, longitudinal analysis only showed an increase in IL-1b during pregnancy. In experimental models, it has been reported that obesity and excessive nutrition alters the inflammatory response, with increasing concentrations in TNF- α, IL1b, and IL-6, among others, resulting in the development of insulin resistance and excessive fetal growth [34]. Higher maternal BMI has also been associated with higher levels of cytokines and the activation of placental inflammatory pathways. In a recent study, the activation of placental inflammatory pathways p38MAPK and STAT3 occurred with increasing maternal BMI and also showed that TNFα activated these pathways in cultured primary human trophoblast cells. Interestingly, placental p38-MAPK activity was correlated with birthweight [15]. However, these mechanisms have not been explored separately in different BMI categories at the beginning of pregnancy.

Excessive gestational weight gain reflects higher fat accretion and has been related with adverse perinatal outcomes [35]. In our study, women who were classified with excessive gestational weight gain showed higher circulating insulin on the third trimester, as reported before [36].

One of our studies´ strengths is the description of metabolic and inflammatory markers in normal weight and overweight/obese women with non-complicated pregnancies (without GDM, preeclampsia, or macrosomia in their newborns). This is relevant given the role that these markers have in modifying the risk of developing GDM [37,38], preeclampsia [39,40] and adiposity programming in offspring [5,12,41].

Given the lack of clinical chart information and the inability to weigh women before pregnancy, self-reporting of pregestational weight is a study limitation. This measurement introduced bias on how women were classified according to BMI category, and thus there exists the possibility that BMI may not modify the effect. Also, given the highly selected profile of the women included in this study, it is not representative of all Mexican women.

In conclusion, maternal weight status altered the concentrations of insulin, leptin, adiponectin, TG and CRP throughout pregnancy. The role of early leptin and TNFα in fetal growth should be studied further, given the association was only observed in women of normal weight. This study presents data distribution of metabolic and inflammatory markers of normal weight and overweight/obese women that did not develop GDM, preeclampsia nor macrosomia.

## Supporting information

S1 Dataset (SAV)

S2 Dataset (SAV)
